# Crystal structure of bis­(4-allyl-2-meth­oxy­phen­yl) terephthalate

**DOI:** 10.1107/S1600536814021229

**Published:** 2014-09-30

**Authors:** Sung Kwon Kang, Byung Hee Han

**Affiliations:** a Department of Chemistry, Chungnam National University, Daejeon 305-764, Republic of Korea

**Keywords:** crystal structure, terephthalate, tyrosinase inhibitors

## Abstract

The asymmetric unit of the title compound, C_28_H_26_O_6_, contains one half-mol­ecule, with the complete molecule generated by a crystallographic inversion center. The central terephthalate and meth­oxy­benzene rings are approximately perpendicular, making a dihedral angle of 80.31 (5)°. No specific directional contacts are noted in the crystal packing. The terminal vinyl group is disordered over two orientations with an occupancy ratio of 0.796 (4):0.204 (4).

## Related literature   

For general background to tyrosinase, see: Ha *et al.* (2007 ▶). For the development of tyrosinase inhibitors, see: Battaini *et al.* (2000 ▶); Thanigaimalai *et al.* (2010 ▶); Cabanes *et al.* (1994 ▶). For the structures of related compounds, see: Choi *et al.* (2011 ▶, 2012 ▶).
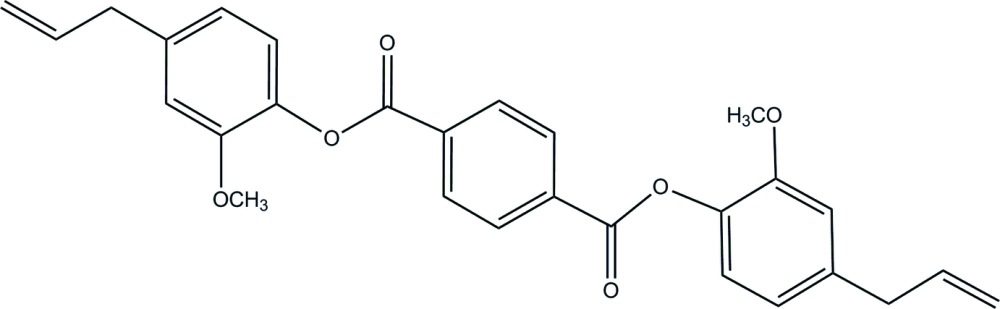


## Experimental   

### Crystal data  

C_28_H_26_O_6_
*M* *_r_* = 458.49
Triclinic, 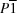

*a* = 7.8853 (2) Å
*b* = 9.0404 (3) Å
*c* = 9.4801 (3) Å
α = 70.660 (4)°
β = 73.817 (3)°
γ = 75.145 (2)°
*V* = 602.28 (4) Å^3^
*Z* = 1
Mo *K*α radiation
μ = 0.09 mm^−1^
*T* = 296 K
0.15 × 0.14 × 0.12 mm

### Data collection  

Bruker SMART CCD area-detector diffractometer
21083 measured reflections
3008 independent reflections
1909 reflections with *I* > 2σ(*I*)
*R*_int_ = 0.050

### Refinement  

*R*[*F* ^2^ > 2σ(*F* ^2^)] = 0.051
*wR*(*F* ^2^) = 0.173
*S* = 1.09
3008 reflections
161 parameters
H-atom parameters not refined
Δρ_max_ = 0.28 e Å^−3^
Δρ_min_ = −0.28 e Å^−3^

### 

Data collection: *SMART* (Bruker, 2002 ▶); cell refinement: *SAINT* (Bruker, 2002 ▶); data reduction: *SAINT*; program(s) used to solve structure: *SHELXS2013* (Sheldrick, 2008 ▶); program(s) used to refine structure: *SHELXL2013* (Sheldrick, 2008 ▶); molecular graphics: *ORTEP-3 for Windows* (Farrugia, 2012 ▶); software used to prepare material for publication: *WinGX* (Farrugia, 2012 ▶).

## Supplementary Material

Crystal structure: contains datablock(s) global, I. DOI: 10.1107/S1600536814021229/tk5342sup1.cif

Structure factors: contains datablock(s) I. DOI: 10.1107/S1600536814021229/tk5342Isup2.hkl

**Fig. 1.**
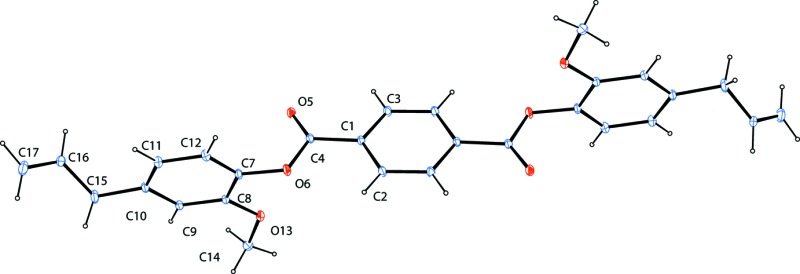
Molecular structure of the title compound, showing the atom-numbering scheme and 30% probability ellipsoids. Only major components of the disordered vinyl group are shown. Unlabeled atoms are related by -x, -y, 1-z]

Supporting information file. DOI: 10.1107/S1600536814021229/tk5342Isup3.cml

x y z . DOI: 10.1107/S1600536814021229/tk5342fig1.tif
Mol­ecular structure of the title compound, showing the atom-numbering scheme and 30% probability ellipsoids. Only major components of the disordered vinyl group are shown. Unlabeled atoms are related by −*x*, −*y*, 1-*z*]

CCDC reference: 1025706

Additional supporting information:  crystallographic information; 3D view; checkCIF report

## supplementary crystallographic information

### S1. Synthesis and crystallization

Terephthaloyl chloride and 4-allyl-2-meth­oxy­phenol were purchased from Sigma Chemical Co. Solvents used for organic synthesis were redistilled before used. All other chemicals and solvents were of analytical grade and used without further purification. The title compound was prepared from the reaction of terephthaloyl chloride (0.203 g, 1 mmol) and 4-allyl-2-meth­oxy­phenol (0.378 g, 2.3 mmol) in tri­ethyl­amine (8 ml) as a solvent. After stirring for 8 h at 333 K under nitro­gen, the reaction mixture was quenched with water and extracted with ethyl acetate. After drying over anhydrous calcium chloride, the solvent was removed by rotary evaporation. The crude product was purified by column chromatography on silica gel using di­chloro­methane: ethyl­acetate (2:1,*v*/*v*) as an eluent. The solution was evaporated to give the product of 0.32 g (70%). Crystals were obtained by slow evaporation from its solution in ethyl alcohol at room temperature.

### S2. Refinement

All H atoms were positioned geometrically and refined using a riding model, with C—H = 0.93-0.97 Å, and with *U*_iso_(H) = 1.5*U*_eq_(C) for methyl, and 1.2*U*_eq_(C) for all other H atoms. The vinyl group is disordered over two positions with an occupancy ratio of 0.796 (4):0.204 (4).

### Crystal data

**Table:**

C_28_H_26_O_6_	*Z* = 1
*M**_r_* = 458.49	*F*(000) = 242
Triclinic, *P*1	*D*_x_ = 1.264 Mg m^−^^3^
Hall symbol: -P 1	Mo *K*α radiation, λ = 0.71073 Å
*a* = 7.8853 (2) Å	Cell parameters from 5115 reflections
*b* = 9.0404 (3) Å	θ = 2.3–26.7°
*c* = 9.4801 (3) Å	µ = 0.09 mm^−^^1^
α = 70.660 (4)°	*T* = 296 K
β = 73.817 (3)°	Block, colourless
γ = 75.145 (2)°	0.15 × 0.14 × 0.12 mm
*V* = 602.28 (4) Å^3^	

### Data collection

**Table:**

Bruker SMART CCD area-detector diffractometer	*R*_int_ = 0.050
Radiation source: fine-focus sealed tube	θ_max_ = 28.4°, θ_min_ = 2.3°
φ and ω scans	*h* = −10→10
21083 measured reflections	*k* = −12→12
3008 independent reflections	*l* = −12→12
1909 reflections with *I* > 2σ(*I*)	

### Refinement

**Table:**

Refinement on *F*^2^	0 restraints
Least-squares matrix: full	Hydrogen site location: inferred from neighbouring sites
*R*[*F*^2^ > 2σ(*F*^2^)] = 0.051	H-atom parameters not refined
*wR*(*F*^2^) = 0.173	*w* = 1/[σ^2^(*F*_o_^2^) + (0.0939*P*)^2^ + 0.0172*P*] where *P* = (*F*_o_^2^ + 2*F*_c_^2^)/3
*S* = 1.09	(Δ/σ)_max_ < 0.001
3008 reflections	Δρ_max_ = 0.28 e Å^−^^3^
161 parameters	Δρ_min_ = −0.28 e Å^−^^3^

### Special details

**Table:**

Geometry. All e.s.d.'s (except the e.s.d. in the dihedral angle between two l.s. planes) are estimated using the full covariance matrix. The cell e.s.d.'s are taken into account individually in the estimation of e.s.d.'s in distances, angles and torsion angles; correlations between e.s.d.'s in cell parameters are only used when they are defined by crystal symmetry. An approximate (isotropic) treatment of cell e.s.d.'s is used for estimating e.s.d.'s involving l.s. planes.

### Fractional atomic coordinates and isotropic or equivalent isotropic displacement parameters (Å^2^)

**Table:**

	*x*	*y*	*z*	*U*_iso_*/*U*_eq_	Occ. (<1)
C1	0.13052 (18)	0.06171 (17)	0.37786 (16)	0.0472 (4)	
C2	0.17983 (19)	−0.04861 (19)	0.50781 (18)	0.0573 (4)	
H2	0.3002	−0.0812	0.5133	0.069*	
C3	−0.04832 (19)	0.10990 (19)	0.37058 (17)	0.0571 (4)	
H3	−0.0808	0.1842	0.2832	0.068*	
C4	0.26416 (18)	0.13439 (17)	0.24324 (17)	0.0495 (4)	
O5	0.22778 (14)	0.22924 (14)	0.12885 (12)	0.0638 (3)	
O6	0.43403 (13)	0.08074 (13)	0.26489 (12)	0.0605 (3)	
C7	0.56778 (19)	0.15655 (18)	0.15091 (17)	0.0528 (4)	
C8	0.58804 (18)	0.30400 (19)	0.15298 (16)	0.0510 (4)	
C9	0.72527 (19)	0.37414 (19)	0.04596 (17)	0.0533 (4)	
H9	0.74	0.4734	0.0448	0.064*	
C10	0.8414 (2)	0.2986 (2)	−0.05965 (17)	0.0563 (4)	
C11	0.8181 (2)	0.1522 (2)	−0.0585 (2)	0.0629 (4)	
H11	0.8951	0.1007	−0.1287	0.075*	
C12	0.6796 (2)	0.0814 (2)	0.04764 (19)	0.0606 (4)	
H12	0.6634	−0.0171	0.048	0.073*	
O13	0.47003 (14)	0.36676 (15)	0.26266 (13)	0.0671 (4)	
C14	0.5024 (3)	0.5069 (3)	0.2795 (2)	0.0772 (5)	
H14A	0.4104	0.5401	0.3594	0.116*	
H14B	0.5013	0.5901	0.1852	0.116*	
H14C	0.6174	0.4852	0.3054	0.116*	
C15	0.9940 (3)	0.3774 (3)	−0.1702 (2)	0.0793 (6)	
H15A	0.9572	0.4919	−0.1881	0.095*	
H15B	1.0958	0.345	−0.1214	0.095*	
C16	1.0537 (4)	0.3429 (5)	−0.3182 (3)	0.1076 (12)	0.796 (4)
H16	0.9698	0.3704	−0.379	0.129*	0.796 (4)
C17	1.2197 (6)	0.2748 (5)	−0.3724 (4)	0.0998 (13)	0.796 (4)
H17A	1.3071	0.2457	−0.3147	0.12*	0.796 (4)
H17B	1.2481	0.2565	−0.4679	0.12*	0.796 (4)
C16A	1.1284 (19)	0.308 (2)	−0.2471 (13)	0.1076 (12)	0.204 (4)
H16A	1.2244	0.2457	−0.2032	0.129*	0.204 (4)
C17A	1.131 (2)	0.328 (2)	−0.4139 (18)	0.0998 (13)	0.204 (4)
H17C	1.0342	0.3905	−0.4567	0.12*	0.204 (4)
H17D	1.2291	0.2776	−0.4728	0.12*	0.204 (4)

### Atomic displacement parameters (Å^2^)

**Table:**

	*U*^11^	*U*^22^	*U*^33^	*U*^12^	*U*^13^	*U*^23^
C1	0.0432 (7)	0.0501 (8)	0.0454 (7)	−0.0193 (6)	−0.0051 (6)	−0.0048 (6)
C2	0.0388 (7)	0.0652 (9)	0.0570 (9)	−0.0165 (7)	−0.0099 (6)	0.0022 (7)
C3	0.0470 (8)	0.0636 (9)	0.0493 (8)	−0.0173 (7)	−0.0112 (6)	0.0050 (7)
C4	0.0437 (7)	0.0533 (8)	0.0483 (8)	−0.0176 (6)	−0.0061 (6)	−0.0064 (6)
O5	0.0527 (6)	0.0754 (7)	0.0511 (6)	−0.0245 (5)	−0.0099 (5)	0.0068 (5)
O6	0.0411 (5)	0.0664 (7)	0.0572 (6)	−0.0207 (5)	−0.0047 (5)	0.0078 (5)
C7	0.0397 (7)	0.0609 (9)	0.0469 (8)	−0.0186 (6)	−0.0056 (6)	0.0033 (7)
C8	0.0408 (7)	0.0666 (9)	0.0430 (7)	−0.0167 (6)	−0.0059 (6)	−0.0093 (7)
C9	0.0468 (8)	0.0635 (9)	0.0487 (8)	−0.0217 (7)	−0.0058 (6)	−0.0091 (7)
C10	0.0442 (8)	0.0669 (10)	0.0509 (8)	−0.0198 (7)	−0.0012 (6)	−0.0078 (7)
C11	0.0493 (8)	0.0692 (10)	0.0624 (10)	−0.0157 (7)	0.0028 (7)	−0.0169 (8)
C12	0.0504 (8)	0.0576 (9)	0.0676 (10)	−0.0171 (7)	−0.0050 (7)	−0.0102 (8)
O13	0.0565 (7)	0.0899 (9)	0.0554 (7)	−0.0283 (6)	0.0073 (5)	−0.0253 (6)
C14	0.0706 (12)	0.1002 (14)	0.0715 (12)	−0.0233 (10)	−0.0042 (9)	−0.0409 (11)
C15	0.0622 (11)	0.0902 (13)	0.0771 (12)	−0.0381 (9)	0.0212 (9)	−0.0240 (10)
C16	0.104 (2)	0.185 (3)	0.0448 (15)	−0.100 (2)	0.0031 (13)	−0.0068 (17)
C17	0.112 (3)	0.115 (3)	0.074 (2)	−0.060 (2)	0.0317 (19)	−0.041 (2)
C16A	0.104 (2)	0.185 (3)	0.0448 (15)	−0.100 (2)	0.0031 (13)	−0.0068 (17)
C17A	0.112 (3)	0.115 (3)	0.074 (2)	−0.060 (2)	0.0317 (19)	−0.041 (2)

### Geometric parameters (Å, º)

**Table:**

C1—C3	1.379 (2)	C11—H11	0.93
C1—C2	1.385 (2)	C12—H12	0.93
C1—C4	1.4903 (18)	O13—C14	1.421 (2)
C2—C3^i^	1.3844 (19)	C14—H14A	0.96
C2—H2	0.93	C14—H14B	0.96
C3—C2^i^	1.3844 (19)	C14—H14C	0.96
C3—H3	0.93	C15—C16A	1.257 (15)
C4—O5	1.1937 (17)	C15—C16	1.461 (4)
C4—O6	1.3512 (17)	C15—H15A	0.97
O6—C7	1.4123 (15)	C15—H15B	0.97
C7—C12	1.360 (2)	C16—C17	1.333 (5)
C7—C8	1.390 (2)	C16—H16	0.93
C8—O13	1.3600 (18)	C17—H17A	0.93
C8—C9	1.3843 (18)	C17—H17B	0.93
C9—C10	1.388 (2)	C16A—C17A	1.53 (2)
C9—H9	0.93	C16A—H16A	0.93
C10—C11	1.378 (2)	C17A—H17C	0.93
C10—C15	1.5154 (19)	C17A—H17D	0.93
C11—C12	1.391 (2)		
			
C3—C1—C2	119.99 (12)	C7—C12—H12	120.1
C3—C1—C4	117.51 (13)	C11—C12—H12	120.1
C2—C1—C4	122.49 (13)	C8—O13—C14	117.13 (12)
C3^i^—C2—C1	119.41 (14)	O13—C14—H14A	109.5
C3^i^—C2—H2	120.3	O13—C14—H14B	109.5
C1—C2—H2	120.3	H14A—C14—H14B	109.5
C1—C3—C2^i^	120.59 (14)	O13—C14—H14C	109.5
C1—C3—H3	119.7	H14A—C14—H14C	109.5
C2^i^—C3—H3	119.7	H14B—C14—H14C	109.5
O5—C4—O6	123.12 (12)	C16A—C15—C10	124.7 (6)
O5—C4—C1	124.73 (13)	C16—C15—C10	116.08 (18)
O6—C4—C1	112.14 (12)	C16—C15—H15A	108.3
C4—O6—C7	116.11 (11)	C10—C15—H15A	108.3
C12—C7—C8	121.45 (13)	C16—C15—H15B	108.3
C12—C7—O6	120.02 (14)	C10—C15—H15B	108.3
C8—C7—O6	118.43 (14)	H15A—C15—H15B	107.4
O13—C8—C9	125.43 (14)	C17—C16—C15	124.7 (4)
O13—C8—C7	116.30 (12)	C17—C16—H16	117.6
C9—C8—C7	118.27 (14)	C15—C16—H16	117.6
C8—C9—C10	121.04 (15)	C16—C17—H17A	120
C8—C9—H9	119.5	C16—C17—H17B	120
C10—C9—H9	119.5	H17A—C17—H17B	120
C11—C10—C9	119.29 (14)	C15—C16A—C17A	118.7 (15)
C11—C10—C15	121.73 (15)	C15—C16A—H16A	120.7
C9—C10—C15	118.95 (15)	C17A—C16A—H16A	120.7
C10—C11—C12	120.13 (16)	C16A—C17A—H17C	120
C10—C11—H11	119.9	C16A—C17A—H17D	120
C12—C11—H11	119.9	H17C—C17A—H17D	120
C7—C12—C11	119.82 (16)		

Symmetry code: (i) −*x*, −*y*, −*z*+1.
