# Supplementary figures and images for: Crystal structure of bis­(4-allyl-2-meth­oxy­phen­yl) terephthalate
Source: Acta Crystallogr Sect E Struct Rep Online. 2014 Sep 30;70(Pt 10):o1138. doi: 10.1107/S1600536814021229 (PMC4257157; doi:10.1107/S1600536814021229)

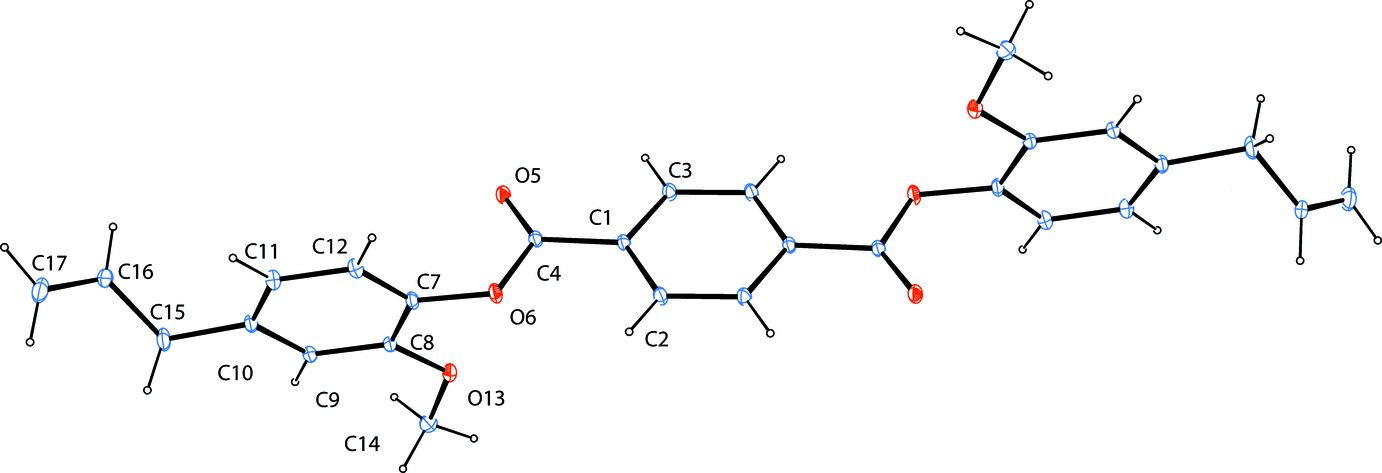

Supplement: Supplementary file 4 [file e-70-o1138-fig1.tif]
